# Development and validation of a Log odds of negative lymph nodes/T stage ratio-based prognostic model for gastric cancer

**DOI:** 10.3389/fonc.2025.1554270

**Published:** 2025-06-03

**Authors:** Haiping Luo, Xiaoming Yu, Xinming Li, Dongzhi Yin, Yugang Cao

**Affiliations:** ^1^ Department of Gastrointestinal Surgery, Huangshi Central Hospital, Affiliated Hospital of Hubei Polytechnic University, Huangshi, China; ^2^ Department of Hepatobiliary Surgery, Huangshi Central Hospital, Affiliated Hospital of Hubei Polytechnic University, Huangshi, China

**Keywords:** T stage, lymph nodes, prognosis, gastric cancer, models

## Abstract

**Purpose:**

To investigate the impact of the ratio of T stage to logarithm of negative lymph nodes (LONT) on the prognosis of gastric cancer patients, and to construct and evaluate CoxPH, RSF, and DeepSurv predictive models for their prognosis.

**Methods:**

A retrospective analysis of clinical, pathological, and prognostic data of patients with gastric cancer from the SEER cohort, TCGA cohort, and GSE62254 cohort was performed. Patients were divided into high-risk and low-risk groups based on the median LONT value. Kaplan-Meier survival curves and log-rank tests were used to compare survival differences between groups. Restricted cubic spline curves, univariate, and multivariate Cox regression analyses were conducted to assess the effect of LONT on patient prognosis. Simultaneously, We sought to develop and validate a novel nomogram based on LONT for predicting overall survival in individual patients with gastric cancer. The performance of the nomogram was evaluated based on the receiver operating characteristic (ROC) curve, calibration curve, and the decision curve analysis (DCA).Weighted gene coexpression network analysis (WGCNA) was used to screened co-expression modules and genes related to LONT, Then Pathway enrichment was performed using Gene Ontology (GO) and Kyoto Encyclopedia of Genes and Genomes (KEGG) analysis for related genes. COX, RSF, and DeepSurv models were constructed using LONT and clinicopathological features to predict overall survival in gastric cancer patients and validated. The predictive performance of these models was evaluated using C-index, time-dependent AUC, and overall Brier score.

**Results:**

In the SEER, TCGA, and GSE62254 cohorts, gastric cancer patients with high LONT expression demonstrated significantly prolonged overall survival compared to those with low expression (P < 0.05). Elevated LONT levels were associated with improved cancer-specific survival in the SEER cohort, disease-specific survival in the TCGA cohort, and disease-free survival in the GSE62254 cohort (P < 0.05). A negative linear relationship between LONT and the hazard ratio for overall survival was observed (P < 0.05), confirming its role as an independent prognostic factor. In the SEER and GSE62254 datasets, LONT outperformed conventional clinicopathological features in predicting overall survival (P < 0.05). The LONT-integrated OS nomogram exhibited robust accuracy, supported by favorable C-index values, well-calibrated plots, and superior net benefit. The CoxPH model surpassed the traditional TNM staging system in discrimination (P < 0.05) while maintaining better calibration than RSF and DeepSurv models. Weighted gene co-expression network analysis (WGCNA) of the TCGA-STAD cohort (soft-threshold power β = 4, R, = 0.92) identified 23 modules, four of which (blue, grey60, red, tan) were strongly correlated with LONT status (|r| > 0.5, P < 0.05). Hub gene screening (|MM| > 0.8, |GS| > 0.1) prioritized 480 genes enriched in focal adhesion, ECM organization, and collagen assembly (P < 0.001, FDR < 0.05). Similarly, WGCNA of the GSE62254 cohort (o = 3, R, = 0.88) revealed three LONT-associated modules (black, blue, cyan; |r| > 0.5, P < 0.05), yielding 111 hub genes. Cross-cohort pathway analysis highlighted dysregulation of cGMP-PKG and relaxin signaling, as well as ECM-integrin interactions. Critically, tumors with low LONT exhibited transcriptional signatures indicative of disrupted ECM homeostasis, providing a mechanistic basis for their aggressive clinical behavior.

**Conclusion:**

LONT is closely related to the overall survival (OS) of gastric cancer patients, and the COX model based on LONT can effectively predict the OS of these patients.

## 1 Introduction

Gastric cancer accounts for 5.6% of all global cancer cases, imposing a significant clinical burden as it ranks fifth in incidence and fourth in mortality among all malignancies. The pTNM staging system has demonstrated robust prognostic stratification and predictive capabilities. However, inadequate sampling of lymph nodes frequently introduces biases in pN staging, raising concerns about the reliability of pTNM staging in assessing gastric cancer prognosis. Despite standardized TNM classifications, substantial variability in patient outcomes persists due to tumor heterogeneity. These limitations necessitate the development of refined risk stratification models to enhance personalized treatment strategies for gastric cancer patients (1). Emerging evidence suggests that the Log odds of negative lymph nodes/T stage ratio (LONT), given its strong association with both tumor pT staging and the number of negative lymph nodes, may serve as a promising indicator for evaluating patient prognosis.

In contemporary clinical practice, various modeling approaches, including Cox proportional hazards, machine learning, and deep learning, have demonstrated distinct advantages in predicting survival outcomes for patients with tumors. However, comparative studies specifically focused on overall survival prediction in gastric cancer patients remain scarce. Many existing models suffer from limited generalizability or insufficient external validation (2). For instance, Alleman et al. highlighted the challenges of applying machine learning models to heterogeneous patient cohorts (3), while Feng et al. emphasized the need for robust external validation in survival analysis studies (4). To address these gaps, we conducted a multi-cohort analysis leveraging SEER, TCGA, and GSE62254 datasets to investigate the prognostic implications of the Log odds of negative lymph nodes/T stage ratio (LONT) in gastric cancer. Furthermore, we established a comprehensive comparative framework that integrates multidimensional clinicopathological variables across three advanced survival prediction models: Cox Proportional Hazards (CoxPH), Random Survival Forests (RSF), and DeepSurv neural networks. This study aims to advance precision prognosis by systematically evaluating the performance of these models and elucidating their potential applications in clinical practice. Through rigorous statistical analyses and cross-validation procedures, our research seeks to provide actionable insights into optimizing survival prediction methodologies for gastric cancer management.

## 2 Materials and methods

### 2.1 Study subjects

Data were obtained from the SEER database (https://www.seer.cancer.gov). Inclusion criteria were: ① Patients diagnosed with gastric cancer by pathology between 2011 and 2016, ICD-O-3 codes C16.0-C16.9; ② Age > 18 years; ③ Complete clinicopathological characteristics (age, gender, T stage, N stage, M stage, number of retrieved lymph nodes, number of positive lymph nodes) and prognostic information (survival time, status). Clinicopathological features and prognostic information from the TCGA and GSE62254 datasets were downloaded from https://portal.gdc.cancer.gov/ and https://www.ncbi.nlm.nih.gov/geo, respectively. Exclusion criteria were: ① Multiple primary cancers; ② Patients with zero survival time; ③ Patients with missing clinicopathological or prognostic information.

### 2.2 Data selection and survival analysis

To construct and validate a broadly applicable model for predicting the prognosis of gastric cancer patients, this study included six clinicopathological features: age, gender, T stage, N stage, M stage, and the logarithm of the ratio of negative lymph nodes to T stage (LONT, where T1, T2, T3, and T4 correspond to 1, 2, 3, and 4, respectively, and LONT = Ln[(number of negative lymph nodes + 1)/T stage]). Survival information included time (in months) and status. Gastric cancer patients were divided into high-LONT and low-LONT groups based on the median value of LONT. The Kaplan-Meier method was employed to estimate the survival rate and plot the survival curves, with the Log-rank test assessing differences between groups. The relationship between LONT and overall survival (OS) in gastric cancer patients was elucidated using restricted cubic spline (RCS) curves. Univariate and multivariate Cox proportional hazards models were utilized to investigate the impact of LONT on the survival of gastric cancer patients.

### 2.3 Construction and validation of the nomogram

The prognostic factors analyzed included age, gender, T stage, N stage, M stage, TNM stage, and LONT were further utilized to construct a nomogram model with the help of R software version 3.3.0 (R Foundation for Statistical Computing, located in Vienna, Austria, accessible via www.r-project.org). Subsequently, the validation and test group were applied to evaluate the newly developed nomogram. The concordance index (C-index) was employed to assess the difference between the predictions made by the nomogram and the actual observed results. A calibration plot was used to visually contrast the prognosis predicted by the nomogram with the real - world outcomes(bootstrap method 1000 times). The receiver operating characteristics curve (ROC) along with the area under the curve (AUC) were used to evaluate the sensitivity and specificity. Moreover, in both the ROC and decision curve analysis (DCA). All these analyses were carried out using R software 3.3.0, and a p - value less than 0.05 was regarded as statistically significant.

### 2.4 Model development and evaluation

Prognostic prediction models for gastric cancer patients based on LONT were constructed using Cox regression, random survival forests (RSF), and deep learning Deepsurv algorithms. The optimal prognostic model was identified through 10-fold cross-validation combined with grid search parameter tuning, followed by predicting overall survival. The predictive performance of each model was evaluated using the concordance index (C-index), mean time-dependent area under the curve (mean time-AUC), and integrated Brier score (IBS).

### 2.5 WGCNA network construction and module identification

Weighted Gene Co - Expression Network Analysis (WGCNA) is a bioinformatics method for studying gene associations across samples. It clusters genes with like expression patterns and explores module - trait relationships. Here, the WGCNA R package built the co - expression network, including genes with adjusted P < 0.05.The process was step - by - step. First, “Hculst” in R did hierarchical clustering to spot outliers. Then, “pickSoftThreshold” chose the right soft thresholding power β for a scale - free network. Next, the “adjacency” function turned the gene expression similarity matrix into an adjacency matrix using β. After that, the adjacency matrix became a topological overlap matrix (TOM) to cut down noise. Finally, hierarchical clustering with the dynamic tree cut function detected modules, and Pearson correlation analyzed module - patient clinical feature correlations (P < 0.05).

### 2.6 Gene enrichment analysis

GO (Gene Ontology) and KEGG (Kyoto Encyclopedia of Genes and Genomes) pathway enrichment analyses were performed using t clusterProfiler package (an R package for comparing biological themes among gene clusters) in R. Enrichment was statistically significant when P < 0.05.

the ECM pathway, including ARHGAP5, DIAPH1, FYN, GSN, HRAS, ITGB1, MAP2K1, MAPK1, MAPK3, MYL2, PFN1, PIK3CA, PIK3CG, PIK3R1, RAF1, RHOA, ROCK1, SHC1 and TLN1 was downloaded from the MSigDB database (https://www.gsea-msigdb.org/gsea/msigdb/) as input files for Gene Set Variation Analysis (GSVA). The GSVA score for gene set across diverse samples was computed using the ssGSEA function implemented in the GSVA package.

### 2.7 Statistical analysis

Data analysis and graphing were performed using Python and R software. Categorical data were described as frequencies (%), analyzed using Pearson chi-square tests or Fisher’s exact tests. Continuous variables were assessed for normality using Shapiro-Wilk tests and for homogeneity of variance using Levene’s test. If the data were normally distributed and variances were homogeneous across groups, Data are expressed as mean ± standard deviation (x ± SD) and compared between two groups using the independent samples t-test. Otherwise, data are presented as median (Median) and interquartile range (IQR, p25-p75), with comparisons between two groups performed using the Mann-Whitney U test. For multiple group comparisons, the Kruskal-Wallis rank sum test was utilized. A P-value <0.05 was considered statistically significant.

## 3 Results

### 3.1 Baseline characteristics

In this study, the SEER cohort included 6806 gastric cancer patients, the TCGA cohort included 341 patients, and the GSE62254 cohort included 300 patients. The median overall survival (OS) for the SEER, TCGA, and GSE62254 cohorts were 36.00 (13.00, 92.00) months, 16.90 (9.20, 27.50) months, and 57.85 (17.84, 78.88) months, respectively, with a statistically significant difference (Z=144.691, P<0.001). The lymph node ratio (LNR) in the SEER, TCGA, and GSE62254 cohorts were 1.79 (0.98, 2.40), 1.47 (0.56, 2.17), and 2.59 (2.01, 2.92), respectively, with a statistically significant difference (Z=205.506, P<0.05). Significant differences were observed across the cohorts for age, T stage, N stage, and M stage (P<0.05). The GSE62254 cohort had the highest proportion of male patients (66%).3%), but the proportion of male patients did not differ significantly across cohorts (P = 0.492), as shown in **Table 1**.

**Table 1:** Baseline characteristics of gastric cancer patients in the SEER, TCGA, and GSE62254 cohorts.

Characteristic	SEER N = 6806 (91%)^1^	TCGA N = 341 (4.6%)^1^	GSE62254 N = 300 (4.0%)^1^	t/Z/χ2	p-value^2^
Status	4,877 (71.7%)	131 (38.4%)	152 (50.7%)	219.570	<0.001
OS(month)	36.00 (13.00, 92.00)	16.90 (9.20, 27.50)	57.85 (17.84, 78.88)	144.691	<0.001
Age	67.00 (57.00, 76.00)	67.00 (58.00, 72.00)	64.00 (55.00, 70.00)	38.345	<0.001
Sex				0.893	0.640
Female	2,429 (35.7%)	127 (37.2%)	101 (33.7%)		
Male	4,377 (64.3%)	214 (62.8%)	199 (66.3%)		
T				390.168	<0.001
T1	1,502 (22.1%)	16 (4.7%)	0 (0.0%)		
T2	3,104 (45.6%)	72 (21.1%)	188 (62.7%)		
T3	1,635 (24.0%)	154 (45.2%)	91 (30.3%)		
T4	565 (8.3%)	99 (29.0%)	21 (7.0%)		
N				247.364	<0.001
N0	2,557 (37.6%)	98 (28.7%)	38 (12.7%)		
N1	2,741 (40.3%)	93 (27.3%)	131 (43.7%)		
N2	1,071 (15.7%)	72 (21.1%)	80 (26.7%)		
N3	437 (6.4%)	78 (22.9%)	51 (17.0%)		
M				6.962	0.031
M0	6,096 (89.6%)	320 (93.8%)	273 (91.0%)		
M1	710 (10.4%)	21 (6.2%)	27 (9.0%)		
LONT	1.79 (0.98, 2.40)	1.47 (0.56, 2.17)	2.59 (2.01, 2.92)	205.506	<0.001

1n (%); Median (IQR) 2Pearson’s Chi-squared test; Kruskal-Wallis rank sum test.

### 3.2 Impact of LONT on OS in gastric cancer patients

**Figure 1:**
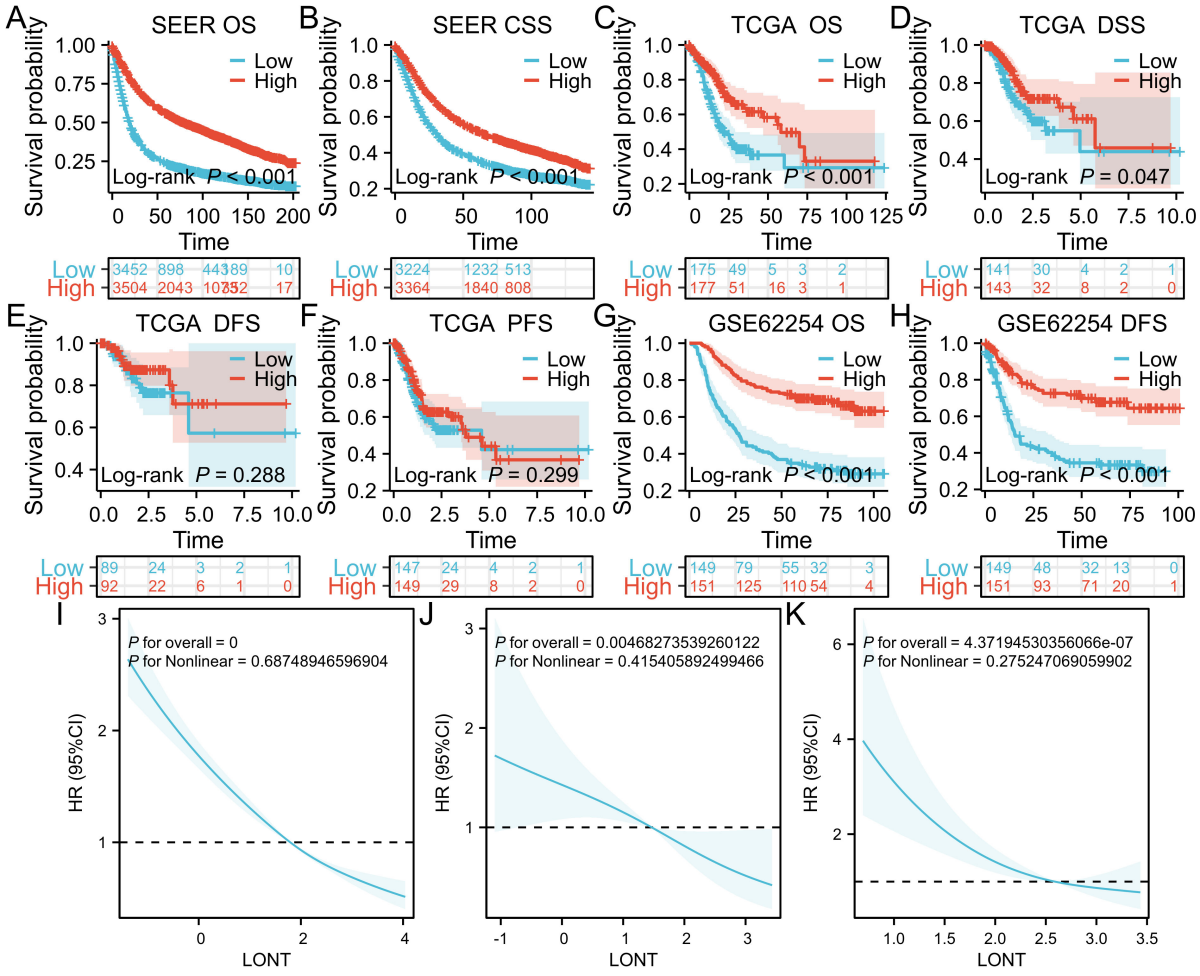
Kaplan-Meier survival curves **(A)** OS in SEER cohort **(B)** CSS in SEER cohort **(C)** OS in TCGA cohort **(D)** DSS in TCGA cohort **(E)** DFS in TCGA **(F)** PFS in TCGA **(G)** OS in GSE62254 cohort **(H)** DFS in GSE62254 cohort **(I)** RCS curves in SEER cohort **(J)** RCS curves in TCGA cohort **(K)** RCS curves in GSE62254 cohort.

In the SEER cohort, high LONT patients had a median overall survival time of 88.00 months (95% CI: 74-88), while low LONT group had an overall survival time of 20.00 months (95%CI: 19-21). The difference between the two groups was statistically significant (HR: 0.455, 95%CI: 0.430-0.482, P<0.05).Significant difference in terms of CSS was observed between the high LONT and low LONT groups (HR: 0.649, 95%CI: 0.611 - 0.689, P<0.05).In the TCGA cohort, high LONT patients had a survival time of 58.23 months (95% CI: 46.9-not reached), while the low LONT group had an overall survival time of 21.73 months (95%CI: 18.3-38.433), with a significant difference observed (HR: 0.509, 95%CI: 0.361-0.718, P<0.05).The mean DSS did differ significantly between the high LONT and low LONT groups (4.96 and 5.75 months, respectively; P=0.04), whereas the mean overall survival time PFS and DFS did not differ (P=0.288 and p=0.299, respectively).In the GSE62254 cohort, the low LONT group had an overall survival time of 27.00 months (95%CI: 21-39.9).The median survival time was not reached in the high LONT group, and the difference was statistically significant (HR: 0.326, 95% CI: 0.236-0.451, P<0.05). Additionally, significant differences were found in the disease-free survival (DFS) time(HR:0.344,95%CI:0.240 - 0.492,P<0.05).After adjusting for age, sex, T stage, N stage, and M stage, the restricted cubic spline (RCS) curve analysis demonstrated a negative linear relationship between gastric cancer LONT and hazard ratio across all cohorts (P<0.05), indicating that increased LONT was associated with reduced mortality risk (**Figure 1**).

### 3.3 Prediction efficacy of LONT on OS status in gastric cancer patients

**Figure 2:**
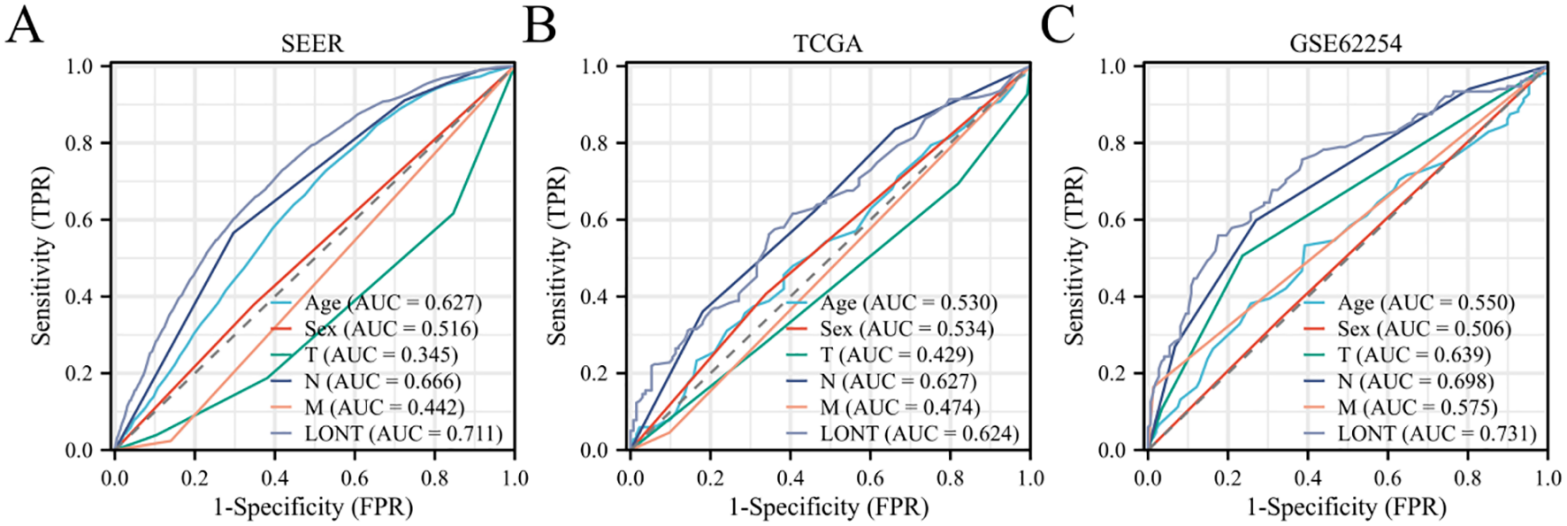
Predictive efficacy of LONT on survival status of gastric cancer patients **(A)** SEER cohort **(B)** TCGA cohort **(C)** GSE62254 cohort.

In the SEER cohort, the AUC value for predicting OS status using LONT was 0.711, whereas the AUC values for age, sex, T stage, N stage, and M stage were 0.627, 0.516, 0.345, 0.666, and 0.442, respectively. The DeLong Z test indicated that the AUC value differences between LONT and these clinicopathological features were statistically significant (P<0.001). In the GSE62254 cohort, LONT’s predictive power for OS status was significantly higher than that of clinicopathological features (P<0.001). In the TCGA cohort, LONT showed superior predictive capability for OS status compared to age, sex, T stage, and M stage (P<0.001).001), but there was no significant difference in predictive power regarding N stage (Z=0.099, P=0.921) (**Figure 2**).

### 3.4 Correlation analysis between clinicopathological characteristics and prognosis in gastric cancer patients

**Figure 3:**
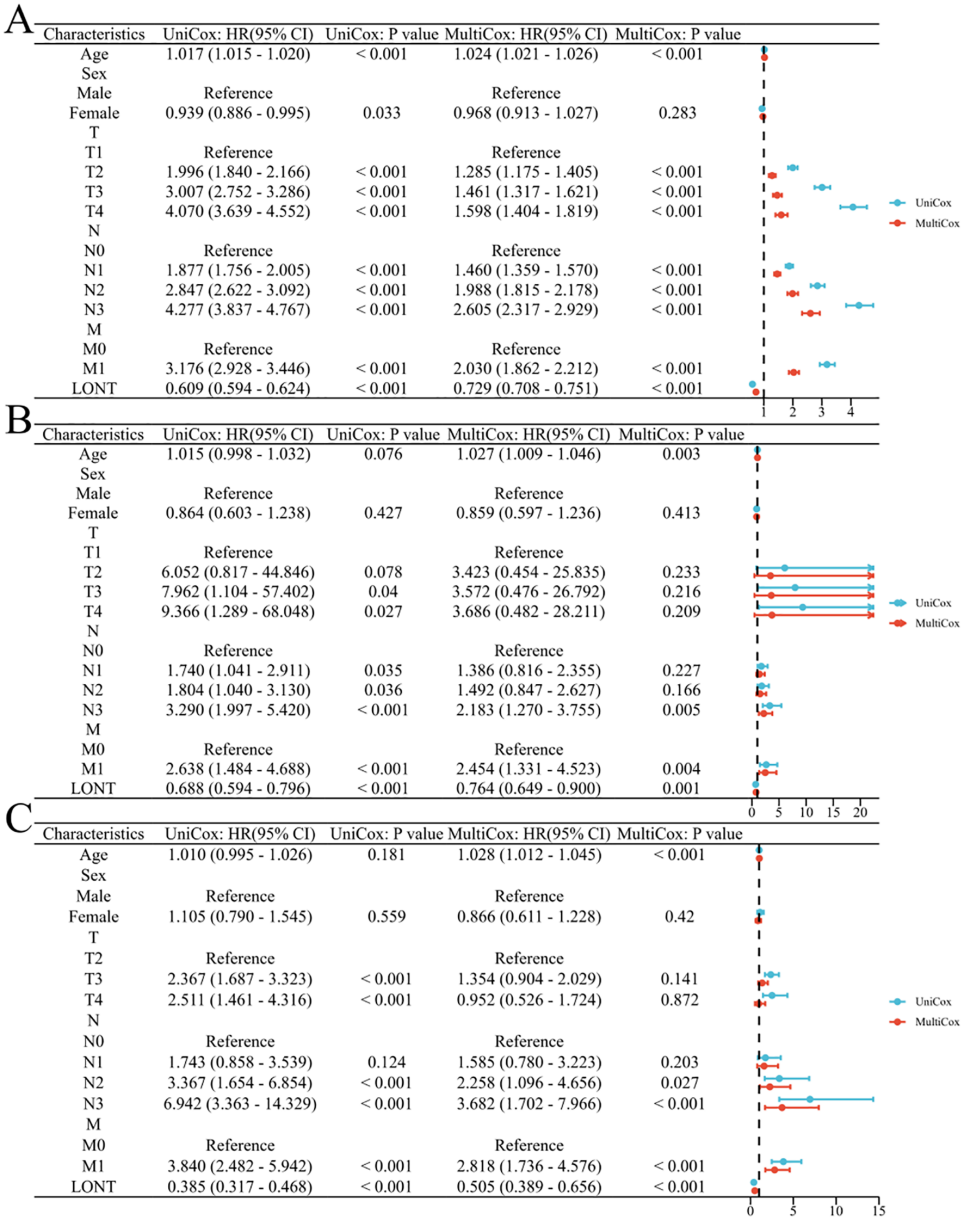
Correlation between clinicopathological characteristics and prognosis in gastric cancer patients **(A)** SEER cohort **(B)** TCGA cohort **(C)** GSE62254 cohort.

In the SEER cohort, univariate COX analysis revealed that age, T2-T4 stages, N1-N3 stages, M1 stage, and LONT were risk factors affecting overall survival (OS) in gastric cancer patients (P<0.05). Multivariate analysis indicated that LONT was an independent risk factor for OS in gastric cancer patients (HR: 0.729, 95% CI: 0.708-0.751, P<0.001). In both the TCGA and GSE62254 cohorts, LONT was identified as an independent prognostic factor for OS in gastric cancer patients (HR: 0.764, 95% CI: 0.649-0.900, P=0.001; HR: 0.505, 95% CI: 0.389-0.656, P<0.001), as shown in **Figure 3**.

### 3.5 Construction and validation of the nomogram

**Figure 4:**
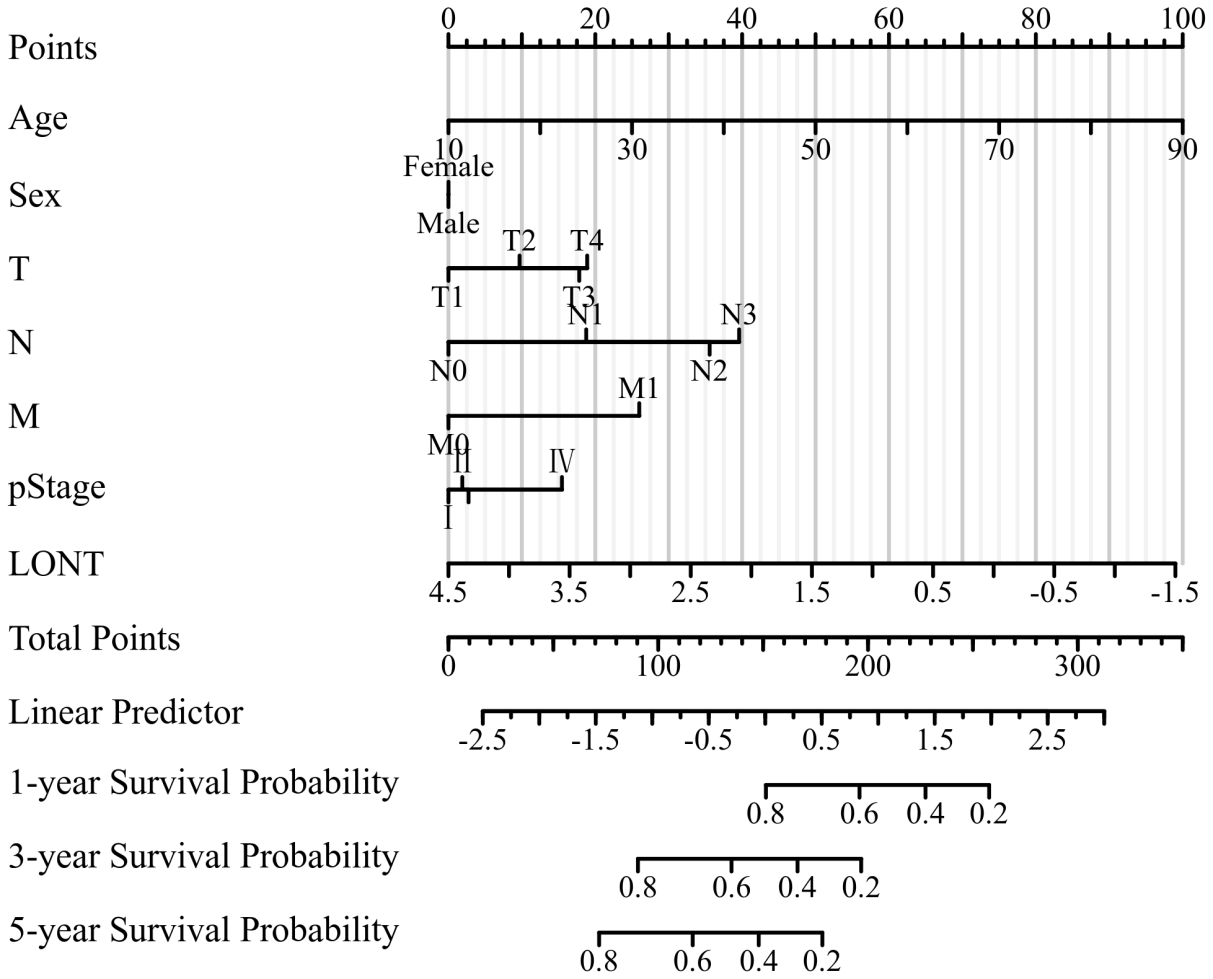
Nomogram predicting 1-, 3-, and 5-year OS of patients with gastric cancer in SEER cohort.

According to the outcomes of clinical significance and univariate Cox analysis result, Age, sex, T stage, N stage, M stage, pstage and LONT were eventually incorporated into final nomogram for predicting OS in the SEER cohort (**Figure 4**). The C-indexes of SEER cohort, TCGA cohort,GSE62254 cohort were 0.707, 0.663 and 0.722 respectively.

**Figure 5:**
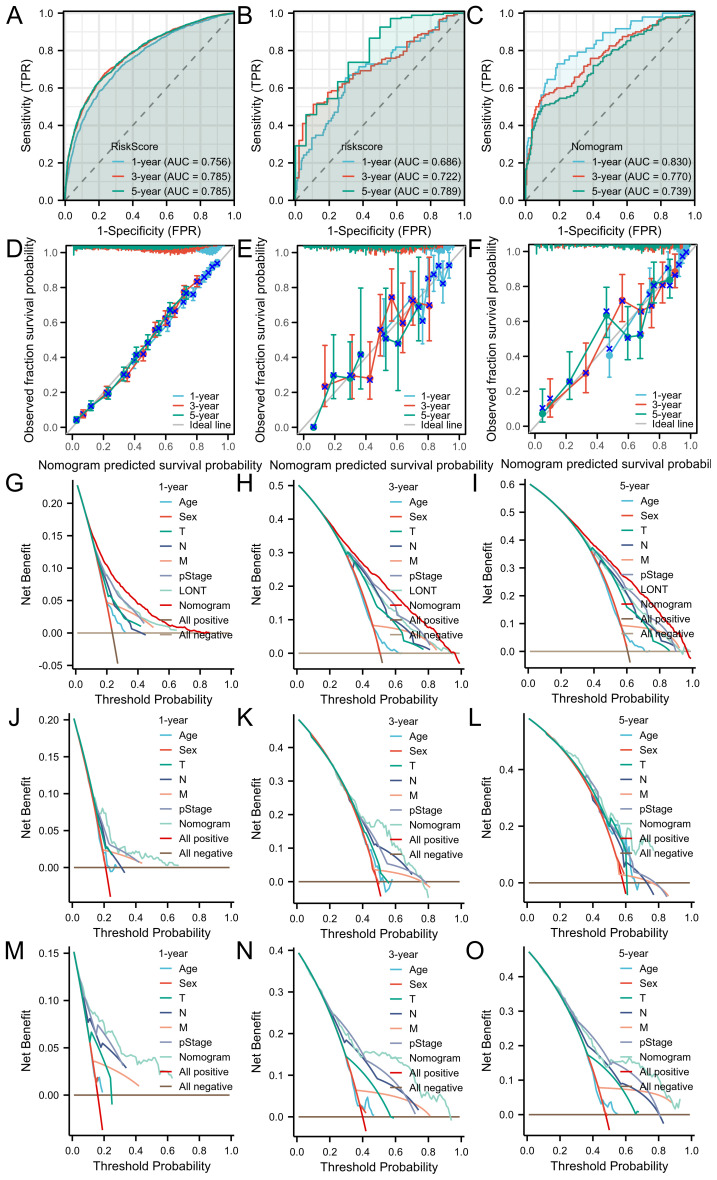
The ROC curves of the nomogram for 1-, 3- and 5-year OS prediction of **(A)** SEER cohort **(B)** TCGA cohort **(C)** GSE62254 cohort. Calibration plots of the nomogram for 1-, 3-year and 5-year OS prediction of **(D)** SEER cohort **(E)** TCGA cohort **(F)** GSE62254 cohort. The nomograms’ DCAs for 1-, 3- and 5-year OS prediction. SEER cohort **(G–I)** and TCGA cohort **(J–L)** and GSE62254 cohort **(M–O)**.

The area under the receiver operating characteristic curve (AUROC) indicated that the model had good stability and discriminative ability. The calibration curve showed that there was a good consistency between the model’s predicted 1-year, 3-year, and 5-year overall survival (OS) of gastric cancer patients in the three cohorts. Decision curve analysis (DCA) demonstrated that the nomogram based on LONT was more effective than univariate analysis in predicting the 1-year, 3-year, and 5-year overall survival (OS) of gastric cancer patients in the SEER, TCGA, and GSE62254 cohorts (**Figure 5**).

### 3.6 Evaluation and comparison of COX, RSF, and DeepSurv models

**Figure 6:**
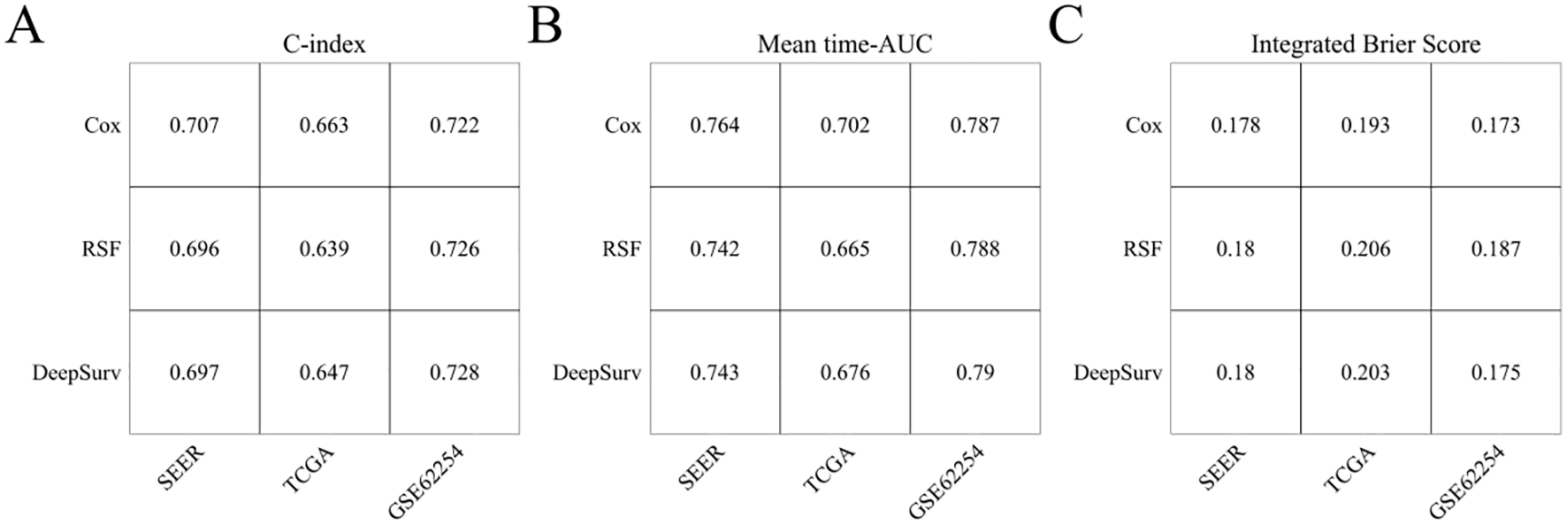
Evaluation of CoxPH, RSF, and DeepSurv models. **(A)** C-index; **(B)** Mean time-AUC; **(C)** Integrated Brier Score.

In the SEER, TCGA, and GSE62254 cohorts, the C-index for TNM staging was 0.662 (95% CI: 0.658-0.666), 0.628 (95% CI: 0.603-0.653), and 0.695 (95% CI: 0.675-0.715). The C-index values of the Cox models incorporating age, sex, T stage, N stage, M stage, and LONT were 0.707 (95% CI: 0.7-0.714), 0.663 (95% CI: 0.608-0.714), and 0.722 (95% CI: 0.68-0.761), respectively. These Cox models demonstrated superior discrimination in predicting overall survival (OS) for gastric cancer patients compared to the conventional TNM staging system (all P<0.001). The C-index and mean time-AUC values of the Cox, RSF, and DeepSurv models were similar across all cohorts, with consistent results, indicating comparable predictive accuracy for OS in gastric cancer patients. All three models had an overall Brier score below 0.25, but the Cox model exhibited the lowest overall Brier score across all cohorts, suggesting better calibration performance than RSF and DeepSurv models. Detailed information is provided in **Figure 6**.

### 3.7 WGCNA and identification of hub genes related with LONT

**Figure 7:**
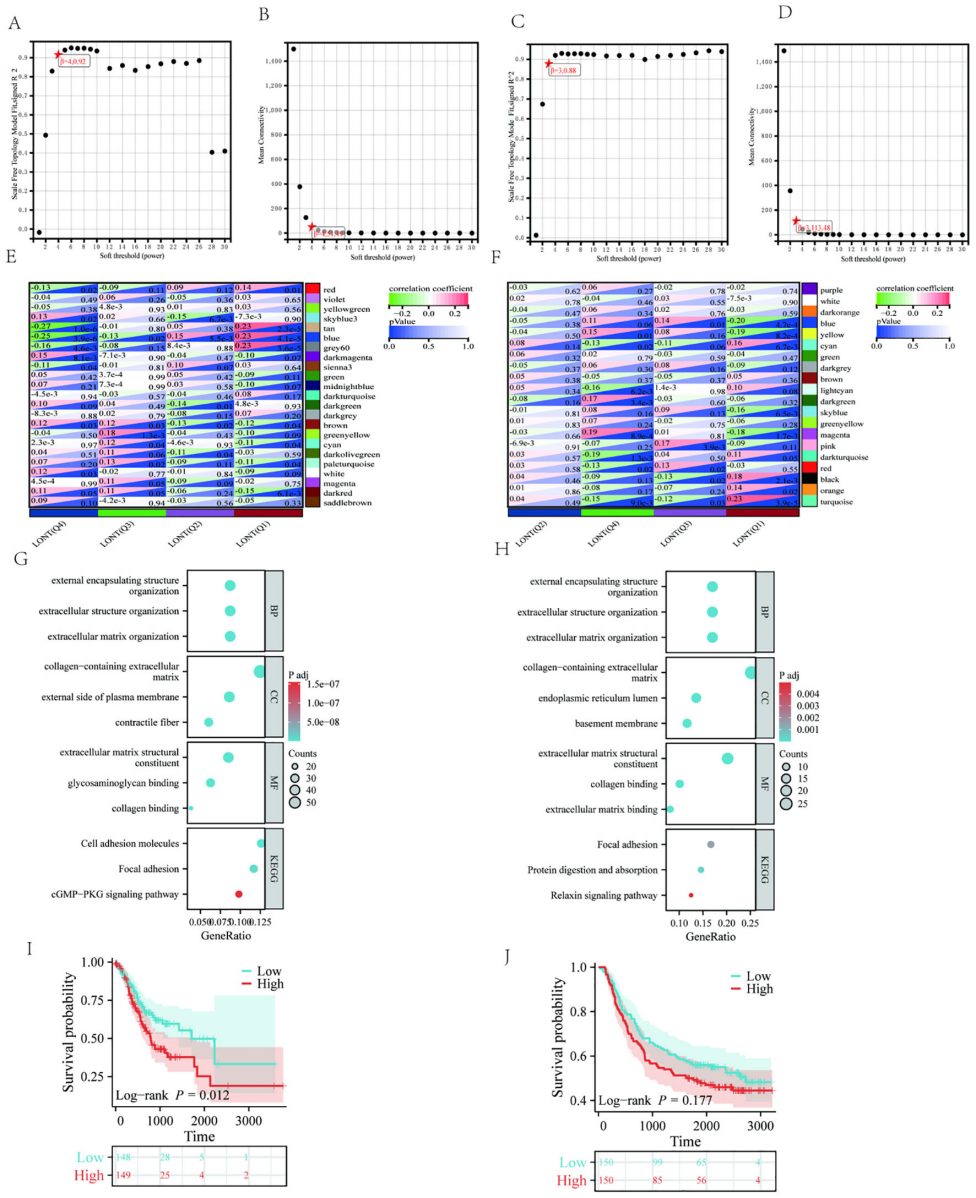
The WGCNA analysis of TCGA and GSE62254 and identification of candidate hub genes **(A)** the soft threshold power in TCGA **(B)** the mean connectivity in TCGA **(C)** A the soft threshold power in GSE62254 **(D)** the mean connectivity in GSE62254 **(E)** The clustered modules of WGCNA in TCGA **(F)** The clustered modules of WGCNA in GSE62254 **(G)** GO and KEEG analysis in TCGA **(H)** GO and KEEG analysis in GSE62254. Kaplan–Meier survival analysis of OS in the different ECM score in **(I)** TCGA cohort **(J)** GSE62254 cohort.

Weighted Gene Co-expression Network Analysis (WGCNA) was applied to the TCGA and GSE62254 datasets. For TCGA, a soft-thresholding power (β = 4, R² = 0.92) generated a scale-free network, yielding 23 modules (**Figures 7A, B**). The blue, grey60, red, and tan modules correlated with LONT (r > 0.5, P < 0.05). Using cutoff criteria (|MM| > 0.8, |GS| > 0.1), 480 hub genes were identified (**Figure 7E**). In GSE62254, β = 3 (R² = 0.88) produced 20 modules (**Figures 7C, D**), with the black, blue, and cyan modules linked to LONT (r > 0.5, P < 0.05). 111 hub genes were selected under the same criteria (**Figure 7F**).

KEGG analysis highlighted enrichment in focal adhesion, cell adhesion molecules, cGMP-PKG signaling, and relaxin signaling pathways. GO analysis implicated these genes in extracellular matrix organization, constituent and collagen banding (**Figures 7G, H**). Moreover,GC patients in the high ECM-score group had a worse OS than those in the low ECM-score group in TCGA cohort (HR: 1.612, 95%CI: 1.107-2.348, P=0.010), except for GSE62254 corhot (HR: 1.245, 95% CI: 0.905-1.713, P=0.177) (**Figures 7I, J**).

## 4 Discussion

The TNM staging system, a cornerstone for evaluating the prognosis of gastric cancer patients, has been widely adopted in clinical practice. Despite advancements in detecting negative lymph nodes (NLNs), which enhance the accuracy of lymph node metastasis assessment, the bias associated with the pN stage due to insufficient detection numbers remains a significant challenge in clinical settings. This limitation complicates the precise stratification of prognostic risks for patients (5). In contrast, LONT (Log Odds of Negative Lymph Nodes/T stage), an innovative prognostic indicator, avoids reliance on the number of positive lymph nodes, effectively mitigating pN stage bias while maintaining a strong correlation with the pT stage (6, 7). Extensive research has demonstrated that LONT not only exhibits substantial prognostic value in bladder cancer and differentiated thyroid cancer but also plays a critical role in predicting outcomes for resectable gastric cancer patients (8).

Moreover, this study confirms that TNM staging serves as an important independent risk factor affecting the overall survival of gastric cancer patients, consistent with previous literature reports (9). The Cox Proportional Hazards Model (CoxPH) constructed based on clinicopathological characteristics and LONT demonstrates superior discrimination ability compared to traditional TNM staging in the SEER, TCGA, and GSE62254 cohorts. In recent years, machine learning and deep learning algorithms have increasingly been applied in the medical field. Models such as Random Survival Forest (RSF) and DeepSurv excel at handling nonlinear variables and high-dimensional data, often outperforming traditional Cox models in prognosis prediction (9–11). For example, RSF shows better performance in terms of calibration, while DeepSurv demonstrates advantages in discrimination (12–14).

However, the findings of this study indicate that the C-index and average time-dependent AUC (time-AUC) values for the COX, RSF, and DeepSurv models are remarkably similar across all analyzed cohorts. These models exhibit comparable discrimination in predicting overall survival (OS) for gastric cancer patients. Notably, the Cox model demonstrates the lowest Integrated Brier Score (IBS), suggesting superior calibration performance compared to both the RSF and DeepSurv models. This observed phenomenon can be attributed to several factors. First, the present study incorporated only six clinicopathological features, which may not fully exploit the advantages of machine learning algorithms such as RSF or deep learning approaches like DeepSurv, particularly in handling complex high-dimensional data. Second, the clinical variables considered herein are readily accessible, and linear regression models, including Cox proportional hazards models, possess inherent advantages such as rapid modeling speed and strong interpretability. Moreover, when applied to multicenter datasets, these models exhibit robust generalizability due to their simplicity and adaptability (6, 8, 14). Consequently, we propose that the COX model based on LONT offers enhanced performance and broader applicability in predicting the overall survival of gastric cancer patients.

Integrative Weighted Gene Co-expression Network Analysis (WGCNA) and functional enrichment analyses revealed that genes associated with low LONT status were significantly implicated in the regulation of extracellular matrix (ECM) homeostasis. Disruption of ECM homeostasis is a well-documented hallmark of cancer, underscoring its critical role in tumor progression and metastasis. Beyond providing structural support for tumor growth, the ECM actively modulates the tumor microenvironment and plays an indispensable role in cancer initiation, development, and progression (15). Based on these insights, we hypothesize that dysregulated ECM homeostasis contributes substantially to the poor prognosis observed in gastric cancer patients with low LONT levels. This hypothesis aligns with established biological principles and highlights the potential mechanistic link between LONT status and ECM-related pathways in gastric cancer pathogenesis.

In summary, LONT represents a critical determinant influencing the overall survival of patients with gastric cancer. The COXPH model developed based on LONT offers an efficient and precise tool for assessing the prognosis of gastric cancer patients. Nevertheless, this study is subject to several limitations that warrant acknowledgment. First, as a retrospective analysis, the relatively limited sample sizes of both the TCGA and GSE62254 cohorts may compromise the external validity of our findings. Second, detailed information regarding surgical techniques and therapeutic regimens was unavailable, thereby precluding a comprehensive evaluation of their potential effects on patient outcomes. Moreover, additional factors known to influence gastric cancer prognosis, such as tumor size, degree of differentiation, and neural or vascular invasion, were not incorporated into the analysis. These omissions underscore the need for further refinement in the comprehensiveness of the model, as previously highlighted in related studies (12, 13, 16).

To enhance the reliability and applicability of LONT-based prognostic assessments, future investigations should focus on validating its predictive capacity across diverse patient populations and clinical settings. Integration of a broader range of clinical variables into the analytical framework will facilitate a more holistic understanding of gastric cancer progression and improve the accuracy of prognostic predictions (17). Such efforts are essential to refine the current model and ensure its utility in guiding personalized treatment strategies for gastric cancer patients.

## Data Availability

The original contributions presented in the study are included in the article/supplementary material. Further inquiries can be directed to the corresponding author.
